# The Utility of Exosomes in Diagnosis and Therapy of Diabetes Mellitus and Associated Complications

**DOI:** 10.3389/fendo.2021.756581

**Published:** 2021-10-26

**Authors:** Yaoxiang Sun, Qing Tao, Xueqin Wu, Ling Zhang, Qi Liu, Lei Wang

**Affiliations:** ^1^ Department of Clinical Laboratory, Yixing People’s Hospital, Yixing, China; ^2^ Center for Translational Medicine and Jiangsu Key Laboratory of Molecular Medicine, Medical School of Nanjing University, Nanjing, China

**Keywords:** exosomes, biomarkers, diabetes mellitus, therapy, diagnosis, mesenchymal stem cells

## Abstract

Diabetes mellitus and the associated complications are metabolic diseases with high morbidity that result in poor quality of health and life. The lack of diagnostic methods for early detection results in patients losing the best treatment opportunity. Oral hypoglycemics and exogenous insulin replenishment are currently the most common therapeutic strategies, which only yield temporary glycemic control rather than curing the disease and its complications. Exosomes are nanoparticles containing bioactive molecules reflecting individual physiological status, regulating metabolism, and repairing damaged tissues. They function as biomarkers of diabetes mellitus and diabetic complications. Considering that exosomes are bioactive molecules, can be obtained from body fluid, and have cell-type specificity, in this review, we highlight the multifold effects of exosomes in the pathology and therapy of diabetes mellitus and diabetic complications.

## 1 Introduction

Exosomes are membranous extracellular vesicles (EVs) first discovered in 1983; for several years, they have been described as organelles removing metabolic waste out of cells (1). Exosomes can be isolated from body fluids such as blood, urine, cerebrospinal fluid (CSF), amniotic fluid, and saliva, and from different cell types *in vitro* such as stem cells, dendritic cells, mast cells, and T cells (2, 3). Recent studies on exosomes extracted from body fluid *in vivo* and culture media *in vitro* have shown that they can provide information about the tissues or cells of their origin and that they act as messengers in cell–cell communication and deliver bioactive molecules such as proteins and nucleic acids, apart from removing cellular waste (4, 5). These studies suggest that exosomes play important roles in non-invasive diagnosis (6) and impaired tissue repair (7).

Diabetes mellitus (DM) is a metabolic disease with high morbidity. It significantly deteriorates the quality of health and life. Early diagnostic methods for diabetes remain lacking, resulting in patients losing the optimal treatment opportunity, which increases the risk of diabetic complications (8). Current therapeutic options include oral hypoglycemic drugs or insulin injections, which provide temporary blood glucose level control; however, these therapies cannot prevent diabetic complications and are associated with adverse effects such as hypoglycemia (9). In this review, we summarize the recent evidence on exosomes as biomarkers and therapeutic factors for DM and its complications in clinical practice.

## 2 Introduction to and Characterization of Exosomes

Exosomes are microvesicles released by cells into the extracellular space, sized around 30–200 nm. Generally, the size of exosomes is at the nanometer level, and the unit for detecting the concentration of exosomes is units/microliter. They can be recognized as a heterogeneous population of membrane-bound structures (“cup-like” or “dish-like”) under a transmission electron microscope (10). Chemical or physical stimulations such as cytokines, unesterified cholesterol, thrombin, tobacco smoke extract (11, 12), hypoxia, and shear stress activate or induce cell apoptosis, which results in the budding of the endosomal membrane, forming multivesicular bodies (MVBs). MVBs and the plasma membrane fuse and finally lead to the release of exosomes (13, 14). Exosomes can be released by most cell types such as cancer cells, stem cells, skeletal muscles cells, mast cells, dendritic cells, and lymphocytes. The most common components in the evaluation of exosomes are the following categories: functional miRNA, a small amount of mRNA, long non-coding RNA (lncRNA), and specific proteins (such as cytokines and growth factors) and other biologically active substances, which are protected from hydrolase activity by the lipid from the original cells and the membrane structures, allowing exosomes to act as cell communication messengers and influence biological function in target cells by fusion, endocytosis, and receptor–ligand interaction (6, 14–17). These are not total components and just one of the components. Exosomes can be extracted from the serum, urine, cerebral spinal fluid, saliva, and bronchiolar lavage fluid (2, 18). The level of exosomes is generally through the detection of their morphology, namely, particle size and concentration. Three main methods are used: morphology (electron microscopy), particle size (diameter particle analysis), and marker protein (WB) (19). Exosome density ranges from 1.13 to 1.21 g/ml, allowing the use of the sucrose-deuteroxide density gradient separation method to isolate them (20). In addition, exosomes can also be extracted using ultra-centrifugation or the ExoQuick exosome precipitation solution (8, 21). CD9, CD63, and CD81 are the accepted surface markers on exosomes for identification using Western blotting (20), quantitative RT-PCR, nucleic acid sequencing, enzyme-linked immunosorbent assay (ELISA), and flow cytometry (FCM).

As exosomes are stable and cell-type specific and can be isolated non-invasively/minimally invasively, they have been extensively studied, particularly in tumorigenesis (22–25) and diagnosis of DM and diabetic complications. Physiologically, exosomes can repair tissue damage, particularly exosomes derived from stem cells (7, 20, 26, 27). Pancreatic, vascular, kidney, nervous, and skin injuries are commonly associated with DM onset and diabetic complications (28–31). Exosomes can physiologically contribute to the repair of such injuries.

## 3 Exosomes as the Potential Biomarkers of Diabetes Mellitus and Diabetic Complications

### 3.1 Introduction to Diabetes Mellitus

DM mainly includes type 1 DM (T1DM) and type 2 DM (T2DM). Under physiological conditions, fasting blood glucose levels should be 3.9–6.1 mM given normal secretion of insulin and tissue insulin sensitivity. Various factors such as genetic inheritance, viral infection, unhealthy lifestyle, and other physical or chemical damages lead to β-cell destruction, impaired insulin secretion, and loss of peripheral tissue insulin sensitivity, finally resulting in a high blood glucose level (28). T1DM accounts for 10% of DM cases and is characterized by absolute insufficiency of insulin, often presenting with symptoms such as thirst, weight loss, and polyuria. T2DM, characterized by insulin resistance in target tissue, relatively insufficient insulin secretion, and subsequent β-cell dysfunction, is often non-symptomatic; and patients with T2DM seek medical care only for complications such as vision loss, heart attack, or limb gangrenes (28, 32). The frequently used diagnostic methods for diabetes include fasted or random blood glucose level measurement for preliminary screening, homeostatic model assessment—insulin resistance (HOMA-IR), oral glucose tolerance tests (OGTTs), intraperitoneal glucose tolerance test for detecting the sensitivity of peripheral tissues to glucose and insulin, serum insulin level, homeostatic model assessment β (HOMA-β) and insulin release tests for determining the function of β cells, and glycated hemoglobin (HbA1c) for indicating the blood glucose level for the previous 8–12 weeks (28, 33).

### 3.2 Introduction to Diabetic Complications

A chronic high blood glucose level disrupts homeostasis, causes oxidant stress, and induces microvessel, nervous, and immune system damage, finally exacerbating the development of diabetic complications (34). Cardiomyopathy is induced by an increased fatty acid metabolism, reduced myofilament Ca^+^ sensitivity, mitochondrial dysfunction, oxidative stress, apoptosis, and fibrosis of diabetic cardiomyocytes (35–37). Pathologic glucose metabolism also damages the blood vessels structurally and functionally, resulting in apoptosis and fibrosis in microvessels, inducing diabetic nephropathy, glomerular atrophy, renal fibrosis, renal dysfunction, and renal failure (29, 38). In the retina, microvessel apoptosis and paraplasm may also result in microaneurysms and hemorrhage, which are diagnosed as diabetic retinopathy and finally cause vision loss (30). Poor glucose control induces peripheral neuropathy and peripheral vascular disease combined with structural deformities, and environmental factors and compromised immunity lead to the development of diabetic foot (31).

Furthermore, heart attack, vision loss, renal dysfunction, and refractory wound healing are often apparent before DM diagnosis, and these symptoms indicate significant organ injury. Therefore, early detection of DM and diabetic complications is crucial; however, no definitive methods of early diagnosis exist (28).

### 3.3 Potential of Exosomes in Non-Invasive/Minimally Invasive Diagnosis

Exosomes are a medium of cell–cell communication and carry several bioactive substances from the original cells including proteins, RNA, DNA, and lipid derivatives (6, 39, 40); they have been studied in both physiological and pathological circumstances such as exercise, cancer, neurodegenerative disorders, and metabolic diseases (41–44). DM and diabetic complications are systemic diseases and affect several organs. The following factors allow the potential use of exosomes in the diagnosis of systemic diseases: 1) exosomes can be derived from the serum, urine, and CSF and contain several bioactive materials like proteins, nucleic acids, and lipids, which can provide information about almost the entire body (14, 45–47). Urine and serum are the common specimen sources of exosomes in DM and associated complication diagnosis; the collection of urine is quite convenient, which can be operated by patients themselves non-invasively; and the collection of blood or CSF is minimally invasive, which can cause no obvious discomfort; 2) exosomes are relatively stable and allow prolonged storage given their membranous structures, which provide structural integrity to bioactive molecules; this feature makes sure of the authenticity and accuracy of results in subsequent tests in that the bilayer structure can avoid the degradation of different kinds of enzyme such as proteolytic enzyme or RNase (48); 3) analysis techniques such as liquid chromatography–mass spectrometry (LC/MS), protein or gene chip analysis, liquid biopsy, FCM, and magnetic bead-based analysis have sufficiently matured to allow using exosomes or tests. The LC/MS can be used to analyze the type and quantity of proteins or metabolites, and genetic sequencing is an important tool of nucleic acid analysis, are which contained in exosomes derived from body fluid of DM or related patients. Besides, Western blotting and qRT-PCR can be used to verify the correlated data in different groups (48–51); 4) several methods for isolation of exosomes exist with acceptable costs (**Table 1**). Ultra-centrifugation is the gold standard method for exosomes isolation, which can promise the highest purity; however, the facility request and operating steps are quite tedious, and the output is quite low; these characteristics result in the low inspection efficiency, which is not clinically applicable (44–46). Sucrose/heavy water density gradient is the improved method of ultra-centrifugation, which increases the output of exosomes, but the steps are still very cumbersome (51). Exosome isolation kit is the most common and convenient method, which has high yield, but the high yield is built on the sacrificing purity (44). In addition, several other methods develop gradually such as magnetic bean sorting, filter device, and flow sorting; in this manners, exosomes can be captured accurately depending on the expensive equipment and consumables and will be the mainstream approach in the future (48, 52–54).

**Table 1:** Methods of exosome isolation and evaluation.

Method	Principle	Advantage	Disadvantage	Reference
Ultra-centrifugation	Special density	Gold standard for vesicle isolation, effective, low cost	Laborious, low yield	(45–47)
Sucrose/heavy water density gradient	Special density	Effective, low cost	Laborious, low yield,	(20, 52)
Exosomes isolation kit	Special density	Convenient, efficient	Low purity and high cost	(45, 47, 50)
Magnetic beans sorting	Immunoreaction	High precision, direct analysis target molecular	Laborious, high cost	(49)
Filter device	Special diameter	High precision, direct analysis of target molecules	High cost	(53)
Flow sorting	Immunoreaction	High precision, direct analysis of target molecule	Laborious, high cost	(54, 55)
PEG (polyethylene glycol)	Special density	Effective	Low purity, high cost	(56)

Herein, we present a review of the recent advances in the use of exosomes as potential early diagnostic biomarkers (**Table 2**) of DM and diabetic complications in different ways, particularly of the recent ones, and the detailed information will be described, as follows.

**Table 2:** Exosomes derived from body fluid can act as novel biomarkers for early diagnosis of DM and diabetic complications.

Disease	Target content in exosome	Sample	Method	Scientific mechanism	Reference
T2DM	Counts of cell derived exosomes ↑	Serum	Flow cytometry meta-analysis	1. Total annexin V-positive blood cell microparticles—procoagulant activity could be involved in vascular complications 2. Endothelial microparticles stimulated by elevated glucose change their molecular composition and increase their biological activity, which may lead to progressive endothelial damage and subsequent cardiovascular complications in diabetes	(57–59)
Diabetes nephropathy	Counts of cell derived exosomes ↑	Urinary	Flow cytometry	MiR-26a-5p from adipose-derived mesenchymal stem cell-derived EVs protect against DN	
	Dipeptidyl peptidase-IV ↑	Urinary	ELISA	The urinary level of microvesicle-bound microvesicle-dipeptidyl peptidase-IV is associated with the severity of diabetic kidney disease	(38)
	Wilms tumor-1 ↑	Urinary	Western blotting	Among podocyte‐derived signal transduction factors in urinary exosomes, WT1 mRNA levels reflected damage of diabetic glomeruli in the patients	(60)
	AMBP, MLL3 ↑VDAC1 ↓	Urinary	LC-MS/MS	Comparing DN urine exosomes and healthy controls, it was discovered in a panel of three proteins (AMBP, MLL3, and VDAC1) that they were differentially found in urinary exosomes from DN patients	(61)
	MiR-130, miR-145, miR-155, miR-424 ↑	Urinary	TaqMan qPCR	High glucose will stimulate mesangial cells and increase the content of miR-145 in mesangial cells and their derived exosomes	(62)
	Mitochondrial DNA ↓	Urinary	Intrarenal Gene Expression Analysis	Urine exosomes from patients with diabetes and CKD had less mitochondrial DNA, and kidney tissues from patients with diabetic kidney disease had lower gene expression of PGC1α	(63)
	Elf3 ↑	Urinary	Western blotting	AGE treatment induced the secretion of Elf3-containing exosomes from cultured podocytes, which was dependent on the activation of the TGF-β-Smad3 signaling pathway	(64)
	MiR-16 ↓	Urinary	RT-qPCR	MiR-16 identified as the most stable endogenous reference gene in data set, making it a suitable endogenous reference gene for miRNA studies of urinary exosomes derived from CKD patients	(65)
	Gelatinase ↓ceruloplasmin ↑	Urinary	ELISA	Gelatinase (decreased activity) and ceruloplasmin (increased levels), in the urinary exosomes of diabetic kidney patients were in agreement with the alterations of these two proteins in the kidney tissue	(66)
Diabetic cardiomyocytes	Counts of exosomes ↑	Blood	Flow cytometry	Exosomes from diabetic rats no longer activated the ERK1/2 and HSP27 cardioprotective pathway and were no longer protective in a primary rat cardiomyocyte model of hypoxia and reoxygenation injury. Exosomes from diabetic plasma have lost the ability to protect cardiomyocytes, but protection can be restored with exosomes from non-diabetic plasma	
	Hsp20 ↓	Serum	LC-MS/MS	Elevation of Hsp20 in cardiomyocytes can offer protection in diabetic hearts through the release of instrumental exosomes	(67)
	MiR-320 ↑	Serum	TaqMan qPCR	Cardiomyocytes exert an anti-angiogenic function in type 2 diabetic rats through exosomal transfer of miR-320 into endothelial cells	(68)
	MiR-126 ↓	Serum	TaqMan qPCR	MiR-126 targets insulin receptor substrate (IRS)-1 expression *via* PI3K/Akt signaling pathways suggests that it is involved in IR modulation	(69)
	MiR-7 ↑	Serum	RT-qPCR	MiR-7 was demonstrated to be involved in β-cell dysfunction and insulin secretion	(70)
Diabetic Charcot neuroarthropathy (CN)	Counts of exosomes ↑	Plasma	Flow cytometry	The concentration of EVs is related to elevation of markers of inflammation (CRP and foot temperature difference) in acute diabetic CN	(71)
Gestational diabetes	Counts of endothelial cell exosomes ↑	Serum, plasma	Western blotting, RT-qPCR	Exosomal Ang2 secretion is regulated by the PI3K/Akt/eNOS and syndecan-4/syntenin pathways	(72, 73)

DM, diabetes mellitus; T2DM, type 2 diabetes mellitus; EV, extracellular vesicle; DN, diabetic nephropathy; LC-MS/MS, liquid chromatography–tandem MS; CKD, chronic kidney disease; AGE, advanced glycation end-product; CRP, C-reactive protein.

#### 3.3.1 Diabetes Mellitus and Diabetic Complications Result in a Change in Exosome Count

The count of exosomes derived from circulating cells differs significantly between those with diabetes and those without, as chronic high glucose levels result in inflammatory cell activation and endothelial cell apoptosis (74). Meta-analyses have revealed a notable increase in circulating exosomes released by platelets, monocytes, and endothelial cells in diabetes; however, exosomes from leucocytes do not differ between patients with diabetes and controls (57, 58). A high glucose concentration can induce a threefold increase in exosomes from endothelial cells (59). A study reported that the count of total exosomes isolated from gingival crevicular fluid of pregnant women who developed gestational DM (GDM) later in pregnancy was significantly higher than in normoglycemic pregnant women (75). In diabetic nephropathy, urinary podocyte exosome counts are higher in patients with T2DM, preceding changes in other biomarkers such as urine albumin or nephrin (an early biomarker of glomerular injury) (76). The exosome counts can be assessed using NanoSight or FCM. Both NanoSight and FCM are experimental methods used to evaluate exosomes. NanoSight technology can detect the size distribution and concentration of purified exosomes through nanoparticle tracking analysis (NTA) (77–79). FCM can be used to observe the number of exosomes and their surface markers (80). It also can be used to identify various exosomal subpopulations (81). The detection of exosomes in the disease through the above techniques may be the most easily accessible method for early screening of DM or diabetes complications.

#### 3.3.2 Differences in Exosome Contents Between Individuals With or Without Diabetes

**Figure 1:**
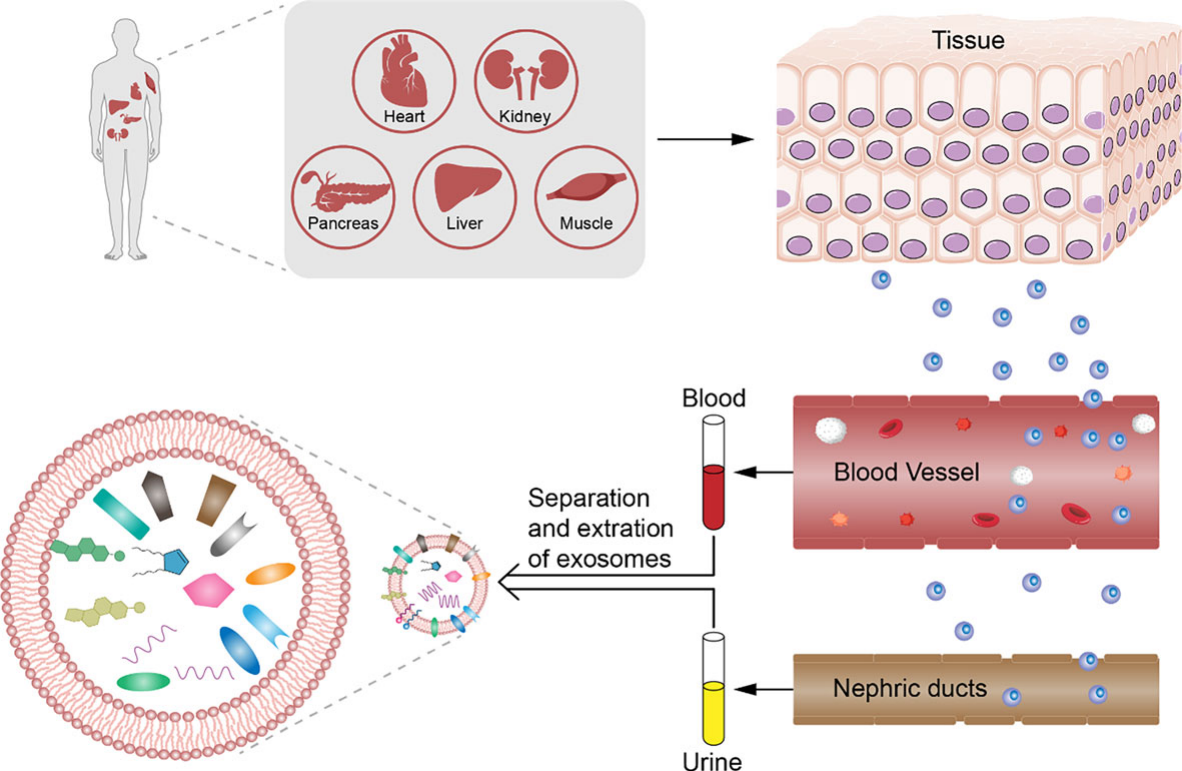
Analysis of exosomes from patients with diabetes mellitus. Diabetes mellitus and diabetic complications have pathological change before organic damage in exosomes derived from body fluids. We can collect the serum, plasma, and urine non-invasively and analyze the counts and contents such as DNA, RNA, and protein depending on the differences between healthy control by choosing the significant biomarker for early diagnosis of diabetes mellitus and diabetic complications.

Although the contents of exosomes between patients with DM have also been reported to vary, the difference is significant in case of diabetic complications. Exosomal proteins derived from body fluids of patients with DM differ; for example, dipeptidyl peptidase-IV (DPP IV) that activates glucagon-like peptide-1 (GLP-1) is associated with DM. In addition, the microvesicle-bound type is the major form of DPP IV in urine, which is significantly higher among those with T2DM than among controls (38). The levels of Wilms tumor protein 1 in urinary exosomes are significantly higher in patients with diabetes with proteinuria, which implies that exosomes can be early biomarkers of podocyte injury (60). The changes in miRNA levels can be detected also in exosomes in diabetes patients with and without diabetic complications. For example, Lange et al. found that the level of miR-16 was lower in the urine of patients with diabetic nephropathy than of healthy controls (65). Individuals with T2DM and T2DM-associated microvascular complications have a significantly higher level of miR-7 in serum-derived exosomes than do individuals without. Accordingly, the changes in these biomarkers in exosomes precede organ-level changes and provide more specificity than whole urine or blood, which further consolidates the potential role of exosomes in early diagnosis of DM and diabetic complications (**Figure 1**).

## 4. Exosomes as a Potential Therapeutic Target for Diabetes Mellitus and Diabetic Complications

### 4.1 The Disadvantages of Traditional Treatment for Diabetes Mellitus and Diabetic Complications

Herein, we focus on common therapeutic strategies for the treatment of DM and its complications (**Table 3**) apart from lifestyle optimizations such as diet control and physical exercise. Insulin injection is the most important therapeutic approach in the treatment of DM and diabetic complications. However, if its dose is not precisely controlled, it can induce life-threatening hypoglycemia (32, 97). Oral hypoglycemic drugs also play key roles in blood glucose level management. Hypoglycemics have been divided into several types depending on their mechanism of blood glucose regulation. For instance, metformin can increase peripheral tissue insulin sensitivity, whereas sulfonylureas can stimulate insulin secretion. Of relevance, these hypoglycemic drugs have adverse effects depending on the mechanism of blood glucose regulation, such as gastrointestinal tract response, hypoglycemia, hypoleucocytosis, hemolytic anemia, increased risk of major cardiovascular events, and weight gain (83–85). There is a correlation between exosomes and traditional diagnostic test results. For example, a study showed that bone marrow mesenchymal stem cell (BM-MSC)-derived exosomes can regulate aging-related insulin resistance. When BM-MSC-derived exosomes are administered to old mice, young wild-type C57/BL6J mouse fasting glucose, fasting serum insulin, and HOMA-IR increased, suggesting that BM-MSC-derived exosomes in old mice can damage the body’s insulin sensitivity (98). Further clinical data showed that after measuring total plasma and EV-related microRNA (miRNA)-15a by real-time PCR, it was found that the circulating levels of miRNA-15a were significantly different. And miRNA-15a has a significant connection with markers of altered glucose metabolism (e.g., HbA1c, plasma glucose, insulin, and HOMA-IR) (99). However, so far, no specific association mechanism between exosomes and HOMA, fasting glucose, OGTT, HbA1c, and other diagnostic indicators has been found.

**Table 3:** The common therapeutic strategies and disadvantages for the DM and its complications.

Disease	Therapy	Treatment principle	Adverse effect	Reference
T1DM	Insulin	Exogenous insulin improves glucose metabolism	Substandard dose control can induce hypoglycemia, ketoacidosis	(82)
T2DM	Insulin	Exogenous insulin improves glucose metabolism	Substandard dose control induces hypoglycemia, ketoacidosis	(82)
	Metformin	Improving peripheral tissue glucose uptake	Gastrointestinal tract response	(83)
	Sulfonylureas/meglitinides	Stimulating insulin secretion	Hypoglycemia, hypoleucocytosis, hemolytic anemia, increased risk of major cardiovascular events, weight gain	(84, 85)
	DPP-4 inhibitors/GLP-1/GIP receptor agonist	Stimulating insulin secretion, suppressing glucagon secretion, slowing gastric emptying, increasing β-cell mass and function	Renal impairment, hypoglycemia	(86–90)
	α-Glucosidase inhibitor	Delaying food decomposition, enhancing GLP-1 secretion	Flatulence, diarrhea	(91–94)
Diabetic cardiomyopathy	Antianginal therapy, percutaneous intervention, surgical revascularization	Reduce myocardial fibrosis, revascularization	Surgical risk, little benefit, other risk factors	(95, 96)

DM, diabetes mellitus; T1DM, type 1 diabetes mellitus; T2DM, type 2 diabetes mellitus.

Currently, effective drugs to treat diabetic complications are lacking, apart from invasive surgery or conventional methods to relieve symptoms such as anti-inflammatory and wound care (95, 96, 100–102).

Glycemic control encompasses the management of not only DM but also the associated complications. However, oral hypoglycemic drugs and insulin can only resolve symptoms and cannot prevent disease development. Therefore, novel strategies for effective treatment, which are non-invasive or minimally invasive with minimal or no adverse effects, are urgently required.

### 4.2 Cell Transplantation for the Treatment of Diabetes Mellitus and Diabetic Complications: Opportunities and Challenges

Promoting insulin secretion and ameliorating insulin resistance are the most important approaches in preventing DM and its complications. Pancreas/islet and stem cell transplantations could be effective and have seen remarkable advancements. We discuss the current research on tissue/cell transplantation for the treatment of DM and diabetic complications.

#### 4.2.1 The Limitations of Pancreas or Islet Transplantation

Pancreas or islet transplantation has been applied in both T1DM and T2DM and has proved successful in restoring functional β cells (103, 104). However, even after several years since first use, these have not been applied extensively for the following reasons. 1) Pancreas or islet transplantation requires surgical intervention. Although islet transplantation is minimally invasive, the risk of portal vein hypertension, thrombosis, or infarction of the liver exists (105–107). 2) Post-transplant autoimmune reactions can cause graft loss and eventual failure (108). 3) To reduce donor antigens, using islets from a single donor (>5,600 islets equivalents/kg) is the best approach; however, this cannot meet the demand, and post-transplant management is complex (109). Patients with glucose lability, insulin resistance, obesity, and donor sensitization are not good candidates for islet transplantation (109). To solve these problems, Sui et al. induced nuclear transfer embryonic stem cells (NT-ES) into C-peptide-positive cells and achieved an average efficiency of 55% *in vitro*, which indicates that this approach could address the challenges of β-cell donation. However, the risk of teratomas remains. Sui et al. (110) found that neuropeptide Y (NPY) family members can activate the Y receptor that inhibits glucagon-like peptide 1 (GLP-1) signaling in β cells and induces insulin secretion. Using Y receptor inhibitors can increase insulin secretion from transplanted islets; however, little is known about Y receptor inhibitors (111), which precludes its immediate extensive clinical use.

#### 4.2.2 The Advantages and Disadvantages of Mesenchymal Stem Cell Transplantation

Currently, MSCs are regarded as a potent regenerative source in repairing injured tissue (26, 50, 112, 113), including in DM and associated diseases. This hypothesis was verified in both animal models and among patients with diabetes. Human umbilical cord MSC (hucMSC) infusion decreased high-fat diet and streptozotocin (STZ)-induced T2DM in rats. Blood glucose level decrease was affected by increasing insulin sensitivity and restoring insulin secretion (114–116). hucMSC injection can also help decrease insulin dependency in patients with T2DM in early stages and hence reduce the insulin dosage at later stages (117). The characteristics of MSCs such as low immunogenicity, proliferation, and multilineage differentiation may partly solve the challenges associated with pancreas or islet transplantation (118, 119). Moreover, genetic editing techniques such as lentivirus and CRISPER/Cas9 in MSCs, which overexpress exendin-4, can be used for pancreatic duodenal homeobox-1-induced MSC differentiation into insulin secretion cells, which may help overcome the shortage in islet donors (120, 121). Besides, MSCs also show remarkable effects in diabetic complications. BM-MSCs seeded in collagen scaffolds can augment angiogenesis in diabetic ulcers in rabbits (122). Placenta-derived MSCs can accelerate foot ulcer repair by inhibiting NF-κB expression and promoting secretion of the anti-inflammatory factor IL-10 (123) in T2DM rat models. In diabetic nephropathy, MSCs derived from several tissues can reverse glomerular injury by inhibiting oxidation, proinflammatory cytokines, and macrophage infiltration (124–127). MSCs also can reverse diabetic neuropathy, cardiopathy, and retinopathy; the underlying mechanism mainly involves improving revascularization, inhibiting fibrosis, controlling inflammation, and regulating oxidation (128–130). Given this body of evidence, MSCs could be the best treatment choice for diabetes and diabetic complications. However, MSC transplantation also presents challenges. First, *in vivo* MSC injection has tumorigenic potential (131–136). Second, the infusion of a large number of MSCs may cause thrombosis (137, 138), headache, and fever (139). Third, the low survival time and efficiency of MSCs *in vivo* may limit their therapeutic efficiency (140). Fourth, although several studies have attempted to improve MSC therapy with techniques such as transfecting CDR1 (141) and hepatocyte nuclear factor-4 alpha (HNF-4α) to regulate the biological characteristics of MSCs directly (142), no practical strategy is applicable in clinical practice and increases the risk of MSC application.

### 4.3 The Advantages of Exosomes in Regulating the Glucose Metabolism in Diabetes Mellitus and Resolving Diabetic Complications

Glucose metabolism regulation by exosomes was first discovered in the setting of physical exercise. Physical exercise is critical in DM care and has proved to increase insulin sensitivity in peripheral tissues and preserve β-cell function (143, 144). Physical exercise or training can also induce rapid release of small EVs from skeletal muscle into circulation, which indicates a connection between exercise-induced exosome release and reversal of insulin resistance and β-cell destruction (145, 146). Furthermore, exosomes released by muscles may contribute to DM management. Glucose-deprived cardiomyocytes released exosomes containing glucose transporter 1 (GLUT1) and GLUT4, and other glucose metabolism enzymes, which can increase glucose uptake and subsequent glycolysis in neighboring endothelial cells (147). Exosomes released during exercise contain miR-455, miR-29b, miR-323-5p, and miR-466, which can downregulate the expression of matrix metalloproteinase (MMP9) by binding to its 3′ region to inhibit MMP9-induced cardiac fibrosis, which may reverse diabetic cardiopathy (15, 148). Previously, our team has used exosomes derived from hucMSCs to treat T2DM rat models, achieved good curative effects in the early stage, and explained the relevant mechanisms (149). In addition, exosomes secreted from INS-1 cells can deliver neutral ceramidase to inhibit palmitic acid (PA)-induced INS-1 cell apoptosis and increase insulin sensitivity in the PA-induced insulin-resistant cell model H4IIEC3 (150). These data showed the potential of physical exercise associated exosomes in regulating glucose metabolism.

Exosomes have also been reported to be effective in the treatment of diabetic complications. For instance, Davidson et al. found that exosomes derived from diabetic rats are not capable of activing the ERK1/2 and HSP27 cardioprotective pathway to protect rat cardiomyocytes from hypoxia and re-oxygenation injury (151); however, exosomes derived from non-diabetic plasma were effective. In addition, human endothelial progenitor cell-derived exosomes contained angiogenesis-related molecules, including FGF-1, VEGFA, VEGFR-2, ANG-1, E-selectin, CXCL-16, eNOS, and IL-8, to accelerate cutaneous wound healing in diabetic rats by improving proliferation, migration, and angiogenic tubule formation in endothelial cells (152); the Erk1/2 signaling pathway was also involved (153). These reports indicate that the contents of exosomes derived from patients with DM or diabetic complications are dysfunctional and incapable of regulating cell–cell communications; however, the use of exogenous exosomes overcomes these limitations.

GDM is the first occurrence or diagnosis of abnormal glucose tolerance during pregnancy; this condition occurs during pregnancy when the pancreatic β-cell function is insufficient to overcome the insulin resistance (154). The incidence of GDM is increasing every year (155). It is associated with various short-term and long-term adverse effects in pregnant women and offspring (156). Early screening and timely intervention are critical to improve the maternal and child outcomes in GDM (157–159). Exosomes can be potential biomarkers for disease diagnosis and early prediction (160, 161), can carry miRNA, lncRNA, protein, and so forth, which act on recipient cells (162) and play a key role in intercellular signal transmission (163). Previous studies have found that in different stages of pregnancy, the levels and biological activities of exosomes in the circulation differ between women with GDM and without (164); however, the miRNA expression changes in exosomes in GDM. The underlying mechanisms are yet to be fully clarified. GDM is associated with proinflammatory processes, oxidative stress, and endothelial cell dysfunction in the placental microvascular system (165). Fetal–placental endothelial dysfunction is characterized by changes in the l-arginine-adenosine signaling pathway and inflammation (165, 166). The mechanisms involved in these changes are hypothesized to be hyperglycemia, hyperinsulinemia, and oxidative stress (167, 168). These conditions increase the release of exosomes. Because exosomes can regulate vascular function, they play an important role in the fetal–placental endothelial dysfunction in pregnancy in women with GDM (165). Increasing evidence shows that miRNAs rich in nanovesicles called exosomes are important regulators of gene expression. Compared with a normal pregnancy, a GDM pregnancy is associated with skeletal muscle insulin resistance and increased levels of circulating placental exosomes. Placental exosomes from women with GDM pregnancy suppressed insulin-stimulated migration and glucose uptake in primary skeletal muscle cells obtained from patients with normal insulin sensitivity. Of interest, placental exosomes from normoglycemic women increased insulin migration and glucose uptake in skeletal muscle of women with diabetes (73).

Although DM and diabetic complications are metabolic diseases, one of the essential causes is tissue injury. For example, auto-antibodies destroy β cells, causing insulin secretion deficiency; lipid mediates activation of macrophages to prominent proinflammatory cytokines and induces insulin resistance (169, 170); chronic high glucose levels and insulin resistance cause increased fatty acid metabolism; the reduced myofilament Ca^+^ sensitivity, mitochondrial dysfunction, oxidative stress, apoptosis, and fibrosis induce endothelial cell apoptosis, cardiomyopathy (30, 31, 34–36), and neuropathy; and chronic high glucose levels and working strength induce glomerular injury and renal fibrosis (29, 76). Based on reported evidence, exosomes can potentially repair tissue injury.

### 4.4 Therapeutic Advantages of Mesenchymal Stem Cell-Derived Exosomes in Diabetes Mellitus and Diabetic Complications

Exosomes can be derived from several tissues and cells; however, exosomes can be derived from MSCs (MSC-ex) most conveniently. MSCs can be isolated from the bone marrow, umbilical cord, and adipose tissue, which can be used in autotransplantation. Low immunogenicity ensures low immunoreactions in such transplantation. The proliferation potential of MScs ensures sufficiency of exosomes.

Currently, the repair of injured tissue by MSCs does not rely on proliferation potential but on paracrine activity, because only <1% MSCs can reach the target tissue, and evidence shows that MSCs differentiated into target cells are lacking (114, 171–173). Exosomes are one of the most important approaches for paracrine regulation. Our previous research showed that exosomes are an excellent replacement for MSCs and played an important role in the repair of injured tissue or organs by delivering bioactive molecules such as Wnt4 (7) and Wnt11 to regulate β-catenin and ameliorate scalded wound, and 14-3-3ζ and glutathione peroxidase 1 to regulate YAP signaling in inhibiting excessive repair and recovering hepatic oxidant injury (8, 50, 112). MSC-ex can also mediate the repair of osteochondral defects by increasing cellular proliferation and infiltration, enhancing matrix synthesis, and a regenerative immune phenotype (174). The present studies not only explain the mechanism underlying MSC-driven repair of tissue injury but also prove that exosomes are key to the paracrine activity of MSCs.

In DM, MSC-ex could be the key element in protecting the pancreatic islets in patients with T1DM from autoimmune targeting, slowing disease progression (175). MSC-ex can promote angiogenesis and survival of transplanted pancreatic islets and can enhance the efficiency and success rate of the treatment, for example, by carrying siFas and anti-miR-375 and inhibiting immune reaction to improve islet transplantation (176, 177). MiR-29b-3p in MSC-derived exosomes significantly ameliorated the insulin resistance in aged mice and helped regulate the blood glucose level (98). Exosomes from the hucMSCs can downregulate blood glucose level in T2DM by reversing peripheral insulin resistance and inhibiting β-cell destruction (149). In diabetic complications, MSC-ex can induce proliferation and migration of normal and chronic wound fibroblasts and enhance angiogenesis to accelerate cutaneous wound healing (178). Diabetes-induced cognitive impairment and nephropathy can be improved by bone marrow stem cell-derived exosomes too (179, 180).

**Figure 2:**
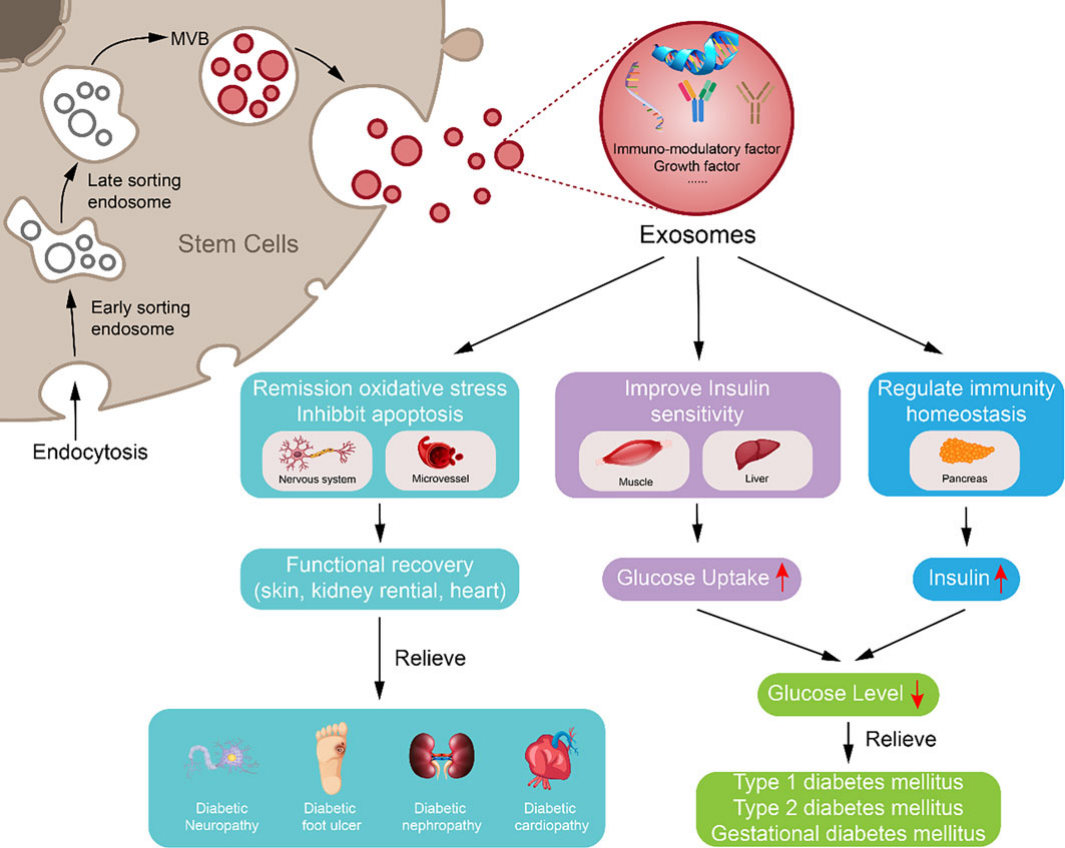
Potential approach of exosomes derived from mesenchymal stem cell in diabetes mellitus and diabetic complication repair. Exosome derived from mesenchymal stem cell may downregulate blood glucose through reversing peripheral tissue (liver and muscle) insulin resistance and increasing β-cell survival during remission of diabetes mellitus; exosome derived from mesenchymal stem cell can inhibit apoptosis, oxidative stress, and immune reaction to reduce vascular or neuron injury and carry growth factors to increase damaged tissue or cell repair, which may participate in alleviating diabetic complications.

Besides ordinary MSC-ex, exosomes from modified MSCs can carry special molecules, like exosomes from 3,3′-diindolylmethane (DIM)-stimulated human hucMSCs contain higher levels of Wnt 11 and enhanced the wound healing potential of hucMSCs (112). Exosomes from hypoxia-inducible factor 1α (HIF-1α) modified BM-MSCs were much more effective in attenuating early steroid-induced avascular necrosis of the femoral head in rabbits than exosomes from the wild-type MSCs (181). These studies indicate that the potency of exosomes can be increased by modifying MSCs, which may be safer than using MSCs directly and can promote the use of exosomes in the treatment of DM and diabetic complications (**Figure 2**).

### 4.5 The Utility of Exosomes in Other Diseases

As a communication messenger between cells, the potential role of exosomes in the clinical treatment and prevention of diseases has gradually emerged. First, in early diagnosis or targeted tumor treatment, various tumor-derived exosomes have been identified that harbor several specific molecules from different types of tumors in patients with cancer (182, 183). In addition, exosomes are associated with neurodegenerative diseases, such as Alzheimer’s disease (AD) and Parkinson’s disease. A recent study found that AD caused by the accumulation of β-amyloid (Aβ) peptides in senile plaques is related to an exosome-associated protein called ALIX, which suggests a significant role of exosomes in the pathogenesis of AD (184). Exosomes can participate in the occurrence and development of cardiovascular diseases. Scientists found that exosomes carrying endothelial differentiation signals affect the formation of new blood vessels, indicating the effectiveness of exosomes in the treatment of angiogenesis (185, 186). In addition, our findings indicated that DIM promoted the stemness of hucMSCs by increasing exosomes derived from hucMSCs to activate Wnt11 autocrine signaling, which provides a novel strategy for improving the therapeutic effects of hucMSCs on skin wound healing (112).

Some studies have shown that exosomes derived from MSCs can also increase ATP levels, reduce oxidative stress through the PI3K/Akt pathway, enhance the vitality of myocardial cells, and prevent adverse remodeling after myocardial ischemia and reperfusion (187). In a study of intervertebral disc degeneration, exosomes can significantly inhibit the inflammatory response of apoptotic nucleus pulposus cells (188).

Exosomes are also involved in the occurrence and development of liver diseases. Karamichali et al. found that exosomes can mediate the transfer of in-frame deletion mutants to regulate HCV virus replication and make the virus continue to infect (189). The concentration of exosomes in the peripheral blood of pregnant women is closely related to the process of pregnancy and pregnancy complications. Abnormal concentration of exosomes in the peripheral blood of pregnant women can reflect the risk of pregnancy complications to a certain extent (190).

### 4.6 The Future Application Prospects of Exosomes in the Treatment of Diabetes and Complications

Nowadays, the application of MSC exosomes is becoming more and more extensive, and the corresponding application technology is relatively mature. In autologous therapy, currently, the main cell-free therapy is MSC-ex. It contains a variety of functional proteins, mRNAs, miRNAs, and signaling lipids (191–193). In non-autologous therapy, researchers are moving towards a new strategy based on loading MSC-ex by patches, injectable microcarriers, or hydrogels, aimed at maintaining the function of exosomes at the function site and enhancing efficiency and safety. Chitosan and relevant compounds are ideal carriers for the sustained release of nanoparticles including exosomes (183, 194, 195). Shi et al. prepared the chitosan/silk hydrogel sponge by freeze-drying method to be a scaffold for exosomes (196). Since chitosan is a hydrophilic polymer, this hydrogel sponge shows good swelling behavior, creates a moist environment, and enhances the angiogenesis and neuronal ingrowth. Alginate-based hydrogels have been designed to encapsulate adipose-derived mesenchymal stem cell exosomes (ADSC-Exos) to fabricate a bioactive scaffold (133), which is tested to be biodegradable and biocompatible, reflecting its potential as a cell-free therapy (197). In general, the exosome-carrier compound displays better treatment outcomes than the exosomes or carrier materials alone, suggesting a synergistic effect through the sustained release of MSC-ex. Not only that, for better delivery of exosomes, MSC exosomes that deliver biological scaffolds have also been invented and used and were fabricated in a 3D-printed cartilage extracellular matrix (ECM)/gelatin methacrylate (GelMA)/exosome scaffold (198).

Besides, in the future, with the help of mass spectrometry and high-throughput sequencing (199, 200), a pathological molecular spectrum of exosomes derived from body fluids of diabetic patients will be formed, covering molecules such as proteins, nucleic acids, and metabolites, which can provide new ideas and research for early diagnosis and prognosis of diabetes. This direction can also provide more options for the treatment of diseases.

## 5 Summary

Exosomes can function not only as biomarkers for early diagnosis of DM but also as potential therapeutic tools in DM and its complications. However, some key challenges exist. The cost of exosome isolation for high volume use is high; no diagnostic and therapeutic standards have been established; and most supporting studies were animal model studies. Further study is needed before extensive clinical use of exosomes can be recommended.

## Author Contributions

YS and QT: conception and design, collection and/or assembly of data, data analysis and interpretation, visualization, manuscript writing, and final approval of the manuscript; these authors contributed equally to this work. XW: collection and/or assembly of data. LZ: collection and/or assembly of data. QL and LW: conception and design, financial support, administrative support, provision of study material, supervision, collection and/or assembly of data, data analysis and interpretation, visualization, manuscript writing, and final approval of the manuscript. All authors reviewed the manuscript. All authors contributed to the article and approved the submitted version.

## Funding

This work was supported by the Natural Science Youth Foundation of the Jiangsu Province (Grant BK20210074), the Introduction program of high-level innovative and entrepreneurial talents in Jiangsu province, Wuxi first “Double hundred” Young and middle-aged Top-notch Medical and health talents Program (HB2020108), Wuxi Health Commission scientific research project youth project (Q202059), and the National Key R&D Program of China (2020YFC2005300).

## Conflict of Interest

The authors declare that the research was conducted in the absence of any commercial or financial relationships that could be construed as a potential conflict of interest.

## Publisher’s Note

All claims expressed in this article are solely those of the authors and do not necessarily represent those of their affiliated organizations, or those of the publisher, the editors and the reviewers. Any product that may be evaluated in this article, or claim that may be made by its manufacturer, is not guaranteed or endorsed by the publisher.
